# Long-Duration Neoadjuvant Therapy with FOLFIRINOX Yields Favorable Outcomes for Patients Who Undergo Surgery for Pancreatic Cancer

**DOI:** 10.1245/s10434-024-15579-0

**Published:** 2024-06-15

**Authors:** Phoebe N. Miller, Fernanda Romero-Hernandez, Lucia Calthorpe, Jaeyun Jane Wang, Sunhee S. Kim, Carlos U. Corvera, Kenzo Hirose, Kimberly S. Kirkwood, Ryutaro Hirose, Ajay V. Maker, Adnan A. Alseidi, Mohamed A. Adam, Grace E. Kim, Margaret A. Tempero, Andrew H. Ko, Eric K. Nakakura

**Affiliations:** 1 Division of Surgical Oncology, Section of Hepatopancreaticobiliary Surgery, University of California, San Francisco, San Francisco, CA USA; 2 UCSF Helen Diller Family Comprehensive Cancer Center, University of California, San Francisco, CA USA; 3 Division of General Surgery, University of California San Francisco, San Francisco, CA USA; 4 Department of Surgery, St. Elizabeth’s Medical Center, Boston, MA USA; 5 Division of Transplant Surgery, Department of Surgery, University of California, San Francisco, San Francisco, CA USA; 6 Department of Pathology, University of California, San Francisco, San Francisco, CA USA; 7 Division of Hematology/Oncology, Department of Medicine, University of California, San Francisco, San Francisco, CA USA

## Abstract

**Background:**

In 2023 alone, it’s estimated that over 64,000 patients will be diagnosed with PDAC and more than 50,000 patients will die of the disease. Current guidelines recommend neoadjuvant therapy for patients with borderline resectable and locally advanced PDAC, and data is emerging on its role in resectable disease. Neoadjuvant chemotherapy may increase the number of patients able to receive complete chemotherapy regimens, increase the rate of microscopically tumor-free resection (R0) margin, and aide in identifying unfavorable tumor biology. To date, this is the largest study to examine surgical outcomes after long-duration neoadjuvant chemotherapy for PDAC.

**Methods:**

Retrospective analysis of single-institution data.

**Results:**

The routine use of long-duration therapy in our study (median cycles: FOLFIRINOX = 10; gemcitabine-based = 7) is unique. The majority (85%) of patients received FOLFIRINOX without radiation therapy; the R0 resection rate was 76%. Median OS was 41 months and did not differ significantly among patients with resectable, borderline-resectable, or locally advanced disease.

**Conclusions:**

This study demonstrates that in patients who undergo surgical resection after receipt of long-duration neoadjuvant FOLFIRINOX therapy alone, survival outcomes are similar regardless of pretreatment resectability status and that favorable surgical outcomes can be attained.

Pancreatic ductal adenocarcinoma (PDAC) has the highest mortality rate of all major cancers, with a 5-year survival rate of 12%.^1^ The American Cancer Society estimates that in 2023 alone, over 64,000 patients will be diagnosed with PDAC and more than 50,000 patients will die of the disease.^2^ Although surgical resection provides the only chance for cure, oncologic outcomes remain dismal. After surgery, adjuvant chemotherapy improves overall survival (OS), and multiagent chemotherapy is associated with improved outcomes relative to gemcitabine alone.^3,4^ However, many patients are unable to complete adjuvant chemotherapy after surgery.^4^

Currently the only US National Comprehensive Cancer Network (NCCN) category 1 recommendations regarding chemotherapy treatment strategies are in the use of adjuvant FOLFIRINOX or gemcitabine-based therapy.^5^ However, many use neoadjuvant therapy for patients with borderline resectable and locally advanced PDAC,^6^ and data are emerging on its role in resectable disease.^7,8^ In addition to possibly increasing the number of patients able to receive complete chemotherapy regimens, neoadjuvant chemotherapy is being used to treat suspected systemic disease, increase the rate of microscopically tumor-free resection margin (R0), identify unfavorable biology, and select patients who are most likely to benefit from surgery.^6^

We previously reported our initial experience with long-duration neoadjuvant FOLFIRINOX (median cycles = 9) for patients (*n* = 26) with borderline resectable disease.^9^ The favorable early results led us to expand the use of long-duration neoadjuvant FOLFIRINOX to patients with resectable and locally advanced disease. We used chemotherapy alone because of the unclear added benefit of radiation therapy to chemotherapy for PDAC in the neoadjuvant and adjuvant setting.^6,7,10–12^ We now report the outcomes of 152 patients, including the 26 reported in our earlier study, treated with long-duration neoadjuvant chemotherapy (FOLFIRINOX *n* = 128, median cycles = 10; gemcitabine-based *n* = 24, median cycles = 7) with resectable (52%), borderline resectable (28%), and locally advanced (20%) disease. Because the vast majority (93%) of patients received chemotherapy only, this study provides important insight into a neoadjuvant treatment approach for PDAC that does not include radiation therapy.

## Methods

### Study Design and Patient Cohort

This was a retrospective analysis of a tertiary care center’s pancreatic cancer surgical database maintained at the University of California, San Francisco (UCSF). Patients were included if they had undergone surgical resection at UCSF for PDAC, received neoadjuvant chemotherapy with or without radiation therapy, and were diagnosed between January 2011 and August 2021. The electronic medical records (EMRs) of individual patients were reviewed and relevant data collected; publicly available records were reviewed to determine the completeness of mortality data. This study was approved by the Committee on Human Research at UCSF.

### Pathologic Evaluation

The primary objective of this study was to determine the rate of R0 resection, defined as the absence of microscopic tumor cells within 1 mm of the surgical margins. On certain pathology reports, this information was not provided and the report of ‘negative margin’ was used (*n* = 8). Secondary objectives were assessments of pathologic, radiographic, and biomarker treatment responses. Pathologic treatment response, estimated by the extent of residual tumor, was graded according to the Evans grading system and was collected from pathology reports. Tumor stage was assessed according to the American Joint Committee on Cancer (AJCC) staging system for pancreatic cancer, with careful consideration for differences between the 7th and 8th edition staging.^13^ Histologic grade, total number of lymph nodes examined, presence of positive lymph nodes, and tumor size were recorded.

### Radiologic Staging

The radiographic effect of neoadjuvant chemotherapy was determined by comparing pre- and post-neoadjuvant therapy imaging. All abdominal multiphasic pancreatic protocol computed tomography (CT) scans obtained at baseline and after chemotherapy and chemotherapy/radiation were reviewed by an experienced surgical oncologist and a radiologist. Tumor location at baseline was recorded. Resectability status, as defined by the NCCN definitions, was used to categorize patients as resectable, borderline resectable (BRPC) and locally advanced (LAPC).^14,15^ In our study, arterial and venous involvement with <180° tumor contact of the vessel circumference was referred to as abutment, and >180° tumor contact was described as encasement. In the case of venous involvement, the presence of vessel narrowing or occlusion was also assessed. Because radiographic measurements according to the Response Evaluation Criteria in Solid Tumors (RECIST) criteria can be challenging for primary pancreatic tumors,^16^ treatment response was qualitatively classified into one of three categories: shrinkage, stable, or progression. Shrinkage was defined as less vascular involvement, evidence of a new fat plane between the tumor and vessel, or decreased primary tumor size; stable was characterized as absence of change in either the primary tumor size or the degree of vascular involvement; and progression was defined as an increase in vascular involvement, new obliteration of a fat plane, and/or increased primary tumor size.

### Neoadjuvant Therapy

Use of and response to neoadjuvant therapy alone (either FOLFIRINOX or gemcitabine-based therapy) or neoadjuvant therapy with radiation therapy were recorded, as were the number of treatment cycles. The total amount of chemotherapy administered was estimated based on the number of treatment cycles reported. One cycle of gemcitabine was a 4-week course with 3 weeks of treatment and 1 week of rest, and of FOLFIRINOX a 2-week course.^17^

### Surgical Resection

Additional study variables included the type of operation, the need for vascular reconstruction, neoadjuvant treatment-related toxicities, perioperative complication rates, frequency and patterns of tumor recurrence, 90-day survival, disease-free survival (DFS), and OS. The Clavien–Dindo complication classification system was used to grade each 30-day surgical complication.^18^ DFS was defined as the time from the date of the first cycle of neoadjuvant chemotherapy until the earliest date of documented disease recurrence or death, and OS was specified as the time from the date of the first cycle of neoadjuvant chemotherapy until the date of death from any cause. Mortality data were collected primarily via EMR review, and publicly available records were reviewed when EMR data were not readily available.

### Statistical Analysis

The Chi-square tests and Mann–Whitney U tests were applied to categorical and continuous variables, respectively. DFS and OS analyses were performed using the Kaplan–Meier method and descriptive statistics were applied to summarize and tabulate the data.

## Results

A total of 152 patients who met the study criteria were identified, of whom 120 received neoadjuvant FOLFIRINOX chemotherapy alone, 8 received neoadjuvant FOLFIRINOX followed by chemoradiation, 22 received gemcitabine-based neoadjuvant therapy alone, and 2 received gemcitabine-based neoadjuvant therapy followed by radiation. Baseline demographic and clinical characteristics are shown in Table 1.

**Table 1:** Patient and tumor characteristics at baseline

	Neoadjuvant FOLFIRINOX alone	Neoadjuvant FOLFIRINOX with RT	Neoadjuvant gemcitabine alone	Neoadjuvant gemcitabine with RT	Overall	*p*-Value
	[*n* = 120]	[*n* = 8]	[*n* = 22]	[*n* = 2]	[*N* = 152]	
Sex						
Male	71 (59)	4 (50)	10 (45)	1 (50)	86 (57)	0.6
Female	49 (41)	4 (50)	12 (55)	1 (50)	66 (44)	
Race						
White	81 (70)	5 (83)	17 (77)	1 (50)	104 (72)	0.7
Black	9 (7.8)	0 (0)	2 (9.1)	0 (0)	11 (7.6)	
Hispanic	8 (7.0)	0 (0)	1 (4.5)	1 (50)	10 (6.9)	
Asian	11 (9.6)	0 (0)	1 (4.5)	0 (0)	12 (8.3)	
Native American	1 (0.9)	0 (0)	0 (0)	0 (0)	1 (0.7)	
Other	4 (3.5)	1 (17)	1 (4.5)	0 (0)	6 (4.1)	
Age at diagnosis, years [median (IQR)]	63 (57, 69)	56 (51, 62)	72 (65, 76)	74 (73, 74)	64 (57, 71)	0.002
Biliary stent placement						
None	52 (43)	4 (50)	6 (27)	1 (50)	63 (41)	0.7
Plastic	26 (22)	1 (12)	4 (18)	1 (50)	32 (21)	
Metal	27 (23)	3 (38)	9 (41)	0 (0)	39 (26)	
Both	9 (7.4)	0 (0)	1 (4.5)	0 (0)	10 (6.5)	
Yes, unknown type	6 (4.9)	0 (0)	2 (9.1)	0 (0)	8 (5.2)	
*Tumor characteristics*						
Tumor location						
Head	65 (54)	6 (75)	15 (68)	1 (50)	86 (57)	>0.9
Uncinate	17 (14)	0 (0)	2 (9.1)	0 (0)	19 (12)	
Head/neck	4 (3.3)	0 (0)	1 (4.5)	0 (0)	5 (3.3)	
Neck	10 (8.3)	1 (12)	1 (4.5)	0 (0)	12 (7.8)	
Neck/body	4 (3.3)	0 (0)	0 (0)	0 (0)	4 (2.6)	
Body/tail	20 (17)	1 (12)	3 (14)	1 (50)	25 (16)	
Tumor type – NCCN						
Resectable	64 (53)	1 (12)	16 (73)	0 (0)	81 (53)	0.005
Borderline resectable	41 (34)	3 (38)	3 (14)	1 (50)	48 (31)	
Locally advanced	15 (13)	4 (50)	3 (14)	1 (50)	23 (15)	
*Vascular involvement (prior to therapy)*						
Superior mesenteric artery						
Abutment	19 (16)	3 (38)	1 (4.5)	1 (50)	24 (16)	0.093
Encasement	5 (4.1)	1 (12)	1 (4.5)	0 (0)	7 (4.5)	
No involvement	96 (80)	4 (50)	20 (91)	1 (50)	121 (80)	
Celiac axis						
Abutment	3 (2.5)	2 (25)	1 (4.5)	0 (0)	6 (3.9)	0.067
Encasement	10 (8.3)	1 (12)	0 (0)	0 (0)	11 (7.2)	
No involvement	107 (89)	5 (62)	21 (95)	2 (100)	135 (89)	
Common hepatic artery						
Abutment	6 (5.0)	0 (0)	2 (9.1)	0 (0)	8 (5)	0.092
Encasement	9 (7.5)	2 (25)	0 (0)	1 (50)	12 (8)	
No involvement	105 (88)	6 (75)	20 (91)	1 (50)	132 (87)	
Superior mesenteric vein						
Abutment	63 (52)	4 (50)	13 (59)	1 (50)	81 (53)	0.2
Encasement	6 (5)	2 (25)	2 (9.1)	1 (50)	11 (7.2)	
Occlusion	3 (2.5)	0 (0)	0 (0)	0 (0)	3 (2)	
No involvement	48 (40)	2 (25)	7 (32)	0 (0)	57 (38)	
Portal vein						
Abutment	14 (12)	2 (25)	1 (4.5)	0 (0)	17 (11)	0.3
Encasement	5 (4.1)	1 (12)	0 (0)	0 (0)	6 (3.9)	
Occlusion	0 (0)	0 (0)	0 (0)	0 (0)	0 (0)	
No involvement	101 (84)	5 (62)	21 (95)	2 (100)	129 (85)	

Data are expressed as *n* (%) unless otherwise specified

*RT* radiotherapy, *IQR* interquartile range, *NCCN* National Comprehensive Cancer Network

According to pretreatment abdominal CT scans, the most common pretreatment tumor location was in the head of the pancreas (*n* = 86, 57%) followed by the neck (*n* = 21, 14%). The most commonly involved vessels were abutment of the superior mesenteric vein (SMV; *n* = 81, 53%), superior mesenteric artery (SMA; *n* = 24, 16%), portal vein (PV; *n* = 17, 11%), and encasement of the common hepatic artery (CHA; *n* = 12, 8%) [Table 1]. When classified by resectability status according to NCCN definitions, most patients who received neoadjuvant chemotherapy alone met formal radiographic criteria for resectable disease (*n* = 81) or BRPC (*n* = 48). For patients with LAPC, FOLFIRINOX alone was the most common regimen (*n* = 15). For the neoadjuvant FOLFIRINOX with radiation cohort, most tumors were classified as BRPC (*n* = 3) or LAPC (*n* = 4) and only one was resectable. Median pretreatment CA19-9 was 181 (range 40–648) and did not vary significantly among neoadjuvant modalities or radiation and non-radiation groups.

The median number of treatment cycles was 10 (range 7–12) for the FOLFIRINOX-alone group and seven (range 6.5–7.5) for the gemcitabine-based-alone group. Overall, 48% of patients showed disease shrinkage and 48% had stable disease on follow-up CT scans (Table 2). Disease shrinkage occurred in three of the eight patients who received FOLFIRONIX and radiation treatment, and one of the two patients who received gemcitabine and radiation treatment.

**Table 2:** Administration of and response to neoadjuvant therapy

	Neoadjuvant FOLFIRINOX alone	Neoadjuvant FOLFIRINOX with RT	Neoadjuvant gemcitabine alone	Neoadjuvant gemcitabine with RT	Overall	*p*-Value
	[*n* = 120]	[*n* = 8]	[*n* = 22]	[*n* = 2]	[*N* = 152]	
No. of treatment cycles [median (IQR)]	10 (7, 12)	9 (8, 10.5)	6 (4, 7.75)	7 (6.5, 7.5)	9 (6, 12)	<0.001
Radiographic response to therapy						
Shrinkage	57 (47)	3 (38)	12 (55)	1 (50)	72 (48)	0.3
Stable	58 (49)	4 (50)	10 (45)	0 (0)	73 (48)	
Progression	3 (2.5)	1 (12)	0 (0)	1 (50)	5 (3.3)	

Data are expressed as *n* (%) unless otherwise specified

*RT* radiotherapy, *IQR* interquartile range

Disease progression occurred in just five patients during neoadjuvant chemotherapy: four in the combined FOLFIRINOX groups and one in the gemcitabine with radiation group. Progression was usually locoregional. Tumor growth resulted in SMA encasement in one patient, SMA and SMV encasement in another, and both PV and SMV encasement in a third (compared with abutment in preoperative scans for all three cases). The fourth patient, whose tumor encased both the CHA and SMV at baseline, received eight cycles of gemcitabine-based neoadjuvant therapy, but the tumor progressed and encased the celiac artery. In the fifth patient, the tumor grew in size but did not encase any vessels. In two of the five cases, an R0 resection was achieved.

Neoadjuvant treatment was shortened or stopped for 40 patients (26.3%) due to various complications, including nausea (22%, *n* = 9), diarrhea and weight loss (15%, *n* = 6), and peripheral neuropathy (1%, *n* = 4). One patient developed hyperbilirubinemia, another developed a hypersensitivity reaction, and another patient developed suppurative cholangitis due to an occluded biliary stent, among others.

Pylorus-preserving pancreaticoduodenectomy was the most common operation performed (49%) (Table 3). Vascular reconstruction was required in 43% of all patients and 47% of those in the FOLFIRINOX-alone group. For all patients in the study group, the 30-day complication rate was 64%, including delayed gastric emptying, wound dehiscence, intra-abdominal abscess, pancreatic fistula, deep vein thrombosis, hypovolemic shock, and urinary tract infection. Clavien–Dindo distribution of grades II, I, and IIIb accounted for 42%, 21%, and 16% of total complications, respectively. Two patients (1.3%) died within 30 days and seven (4.6%) within 90 days postoperatively.

**Table 3:** Operative outcomes and perioperative complications

	Neoadjuvant FOLFIRINOX alone	Neoadjuvant FOLFIRINOX with RT	Neoadjuvant gemcitabine alone	Neoadjuvant gemcitabine with RT	Overall	*p*-Value
	[*n* = 120]	[*n* = 8]	[*n* = 22]	[*n* = 2]	[*N* = 152]	
Type of operation						
Classic PD	31 (26)	2 (25)	4 (18)	1 (50)	38 (25)	0.5
Pylorus-preserving PD	57 (48)	4 (50)	13 (59)	0 (0)	74 (49)	
Distal pancreatectomy	20 (17)	0 (0)	4 (18)	1 (50)	25 (16)	
Celiac axis resection with DP	3 (2.5)	0 (0)	0 (0)	0 (0)	3 (2)	
Extended DP	1 (1)	1 (13)	0 (0)	0 (0)	2 (1)	
Total pancreatectomy	8 (7)	1 (12)	1 (5)	0 (0)	10 (7)	
Vascular reconstruction required	56 (47)	6 (75)	3 (14)	1 (50)	66 (43)	0.003
ASA score						
2	50 (42)	2 (25)	5 (22)	1 (50)	58 (38)	0.3
3	68 (56)	6 (75)	16 (73)	1 (50)	91 (59)	
4	0 (0)	0 (0)	1 (5)	0 (0)	1 (0)	
Clavien–Dindo classification						
I	14 (18)	4 (57)	3 (23)	0 (0)	21 (21)	0.6
II	34 (44)	2 (29)	5 (38)	1 (50)	42 (42)	
IIIa	12 (15)	1 (14)	1 (7.7)	1 (50)	15 (15)	
IIIb	13 (17)	0 (0)	3 (23)	0 (0)	16 (16)	
IVa	0 (0)	0 (0)	1 (7.7)	0 (0)	1 (1)	
V	2 (2.6)	0 (0)	0 (0)	0 (0)	2 (2)	

Data are expressed as *n* (%)

*PD* pancreaticoduodenectomy, *DP* distal pancreatectomy, *RT* radiotherapy, *ASA* American Society of Anesthesiologists

As shown in Table 4, R0 resection was achieved for 74% of all patients and 76% of patients in the FOLFIRINOX-alone group. Of those who received FOLFIRINOX alone, 43% had lymph node involvement and a median of 21 (range 15–27) lymph nodes were examined. The most common AJCC pathologic stages were yIIB (31%) and yIA (18%).

**Table 4:** Histopathologic features and treatment response

	Neoadjuvant FOLFIRINOX alone	Neoadjuvant FOLFIRINOX with RT	Neoadjuvant gemcitabine alone	Neoadjuvant gemcitabine with RT	Overall	*p*-Value
	[*n* = 120]	[*n* = 8]	[*n* = 22]	[*n* = 2]	[*N* = 152]	
Histologic grade						
Well-differentiated	17 (17)	0 (0)	3 (17)	0 (0)	20 (17)	0.8
Moderately differentiated	52 (53)	3 (100)	8 (44)	1 (100)	64 (53)	
Poorly differentiated	29 (30)	0 (0)	7 (39)	0 (0)	36 (30)	
Evans grading system						
I	34 (28)	0 (0)	4 (18)	0 (0)	38 (25)	0.003
II	40 (33)	0 (0)	7 (32)	2 (100)	49 (32)	
III	12 (11)	2 (25)	0 (0)	0 (0)	14 (10)	
IV (no viable tumor cells)	4 (3)	1 (12)	1 (5)	0 (0)	6 (4)	
Unknown	31 (26)	5 (63)	10 (45)	0 (0)	46 (30)	
Invasive tumor size, cm [median (IQR)]	2.10 (1.48, 3.10)	1.90 (0.00, 2.18)	2.95 (1.75, 3.45)	3.65 (3.38, 3.93)	2.10 (1.50, 3.10)	0.065
Surgical margin-negative/R0	91 (76)	7 (88)	15 (68)	0 (0)	113 (74)	0.093
Positive lymph nodes	52 (43)	3 (38)	12 (55)	0 (0)	67 (44)	0.6
Total lymph nodes examined [median (IQR)]	20 (15, 27)	18 (14, 22)	22 (18, 28)	18 (12, 25)	21 (16, 27)	0.7
AJCC/UICC stage						
0	4 (3)	1 (12)	1 (4.5)	0 (0)	6 (4)	
IA	23 (19)	1 (12)	4 (18)	0 (0)	28 (18)	
IB	17 (14)	4 (50)	2 (9)	1 (50)	24 (16)	
IIA	20 (17)	0 (0)	3 (14)	1 (50)	24 (16)	
IIB	39 (32)	1 (12)	7 (32)	0 (0)	47 (31)	
III	17 (14)	1 (12)	5 (23)	0 (0)	23 (15)	

Data are expressed as *n* (%) unless otherwise specified

*RT* radiotherapy, *IQR* interquartile range, *AJCC* American Joint Committee on Cancer, *UICC* Union for International Cancer Control

Ten percent (*n* = 14) of all patients had a treatment response of Evans grade III, corresponding to < 10% of residual tumor cells. Six patients (4%) had a complete response, described as AJCC stage 0 and Evans grade IV (no viable tumor). Fifty percent of patients had no nodal metastasis with AJCC staging between yIA and yIIA.

**Fig. 1:**
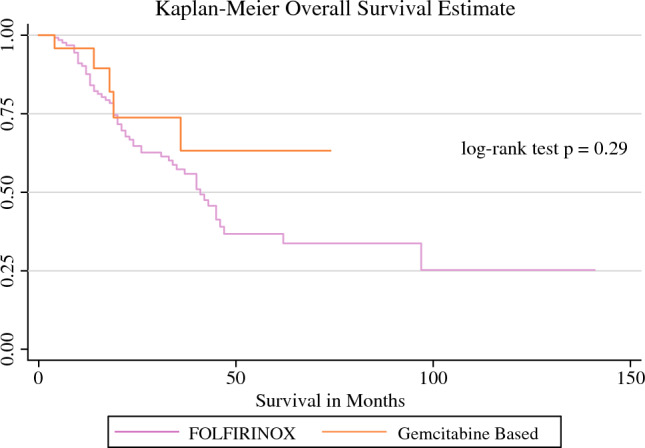
OS of patients who underwent neoadjuvant chemotherapy with and without radiation therapy. The median OS for the FOLFIRINOX groups was 41 months, whereas the median OS for the gemcitabine groups was not reached. *OS* overall survival

**Fig. 2:**
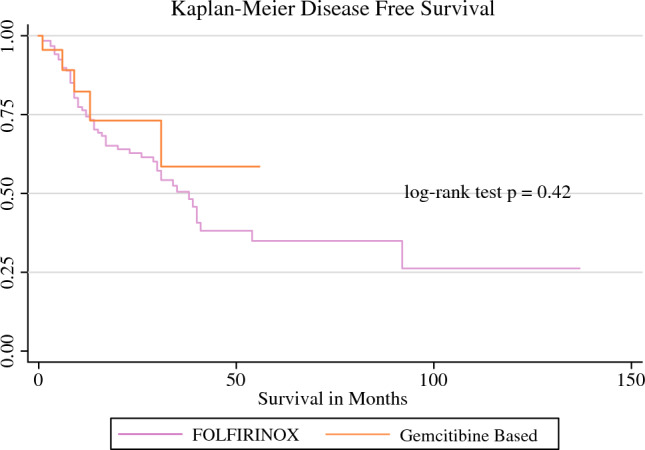
DFS of patients who underwent neoadjuvant chemotherapy with and without radiation therapy. The median DFS in the FOLFIRINOX groups was 38 months, whereas the median DFS for the gemcitabine groups was not reached. *DFS* disease-free survival

**Fig. 3:**
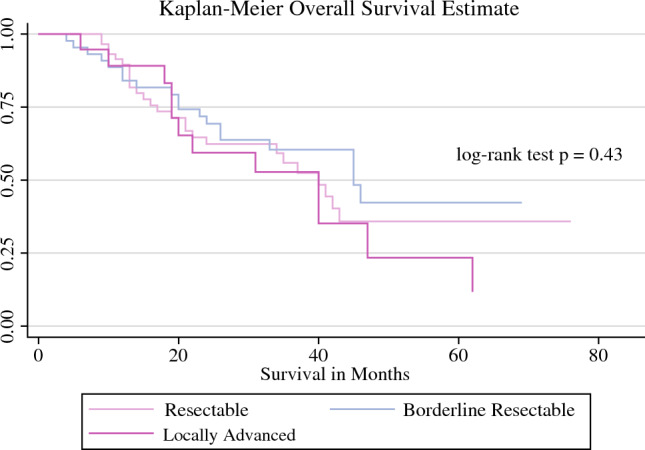
OS of patients who underwent FOLFIRINOX alone (no radiation therapy) stratified by resectability status. The median OS for patients with resectable, borderline resectable, and locally advanced disease was 37 months, 45 months, and 40 months, respectively. *OS* overall survival

**Fig. 4:**
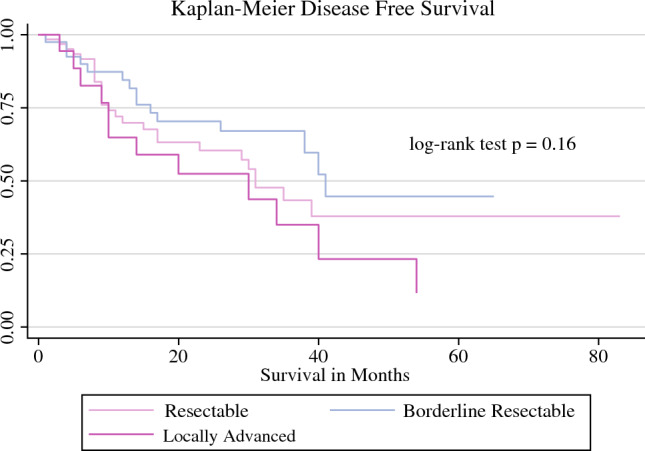
DFS of patients who underwent FOLFIRINOX alone (no radiation therapy) stratified by resectability status. The median DFS for patients with resectable, borderline resectable, and locally advanced disease was 31 months, 41 months, and 30 months, respectively, and the relative hazard ratio was 1.1, 0.74, and 1.5 for resectable, borderline resectable, and locally advanced disease, respectively. *DFS* disease-free survival

The overall recurrence rate of the entire cohort was 45% (Table 5). The median OS for patients who received FOLFIRINOX with or without radiation was 41 months (Fig. 1) and DFS was 38 months (Fig. 2). For patients who received gemcitabine-based therapy with and without radiation, neither the median OS nor median DFS were reached. No difference was seen in OS between patients who received FOLFIRINOX and gemcitabine (Figs. 1, 2). The median OS for resectable disease was 37 months, 45 months for BRPC disease, and 40 months for LAPC (Fig. 3) Median OS and DFS did not significantly differ among patients with resectable disease, BRPC, and LAPC, however BRPC trended towards longer OS and DFS (Fig. 3, 4).

**Table 5:** Postoperative follow-up

	Neoadjuvant FOLFIRINOX alone	Neoadjuvant FOLFIRINOX with RT	Neoadjuvant gemcitabine alone	Neoadjuvant gemcitabine with RT	Overall	*p*-Value
	[*n* = 120]	[*n* = 8]	[*n* = 22]	[*n* = 2]	[*N* = 152]	
90-day survival						
No	5 (4.2)	0 (0)	2 (9)	0 (0)	7 (5)	0.5
Yes	110 (92)	7 (88)	15 (68)	1 (50)	133 (88)	
Recurrence						
No	63 (52)	5 (62)	14 (64)	2 (100)	84 (55)	0.6
Yes	57 (48)	3 (38)	8 (36)	0 (0)	68 (45)	
Death from disease						
No	76 (62)	7 (88)	17 (76)	2 (100)	102 (67)	0.3
Yes	44 (38)	1 (12)	5 (24)	0 (0)	50 (34)	

Data are expressed as *n* (%)

*RT* radiotherapy

## Discussion

To date, this is the largest study to examine surgical outcomes after long-duration neoadjuvant chemotherapy for PDAC. Although neoadjuvant therapy is commonly used for PDAC, the routine use of long-duration therapy (FOLFIRINOX, median cycles = 10; gemcitabine-based, median cycles = 7) is unique. We found that FOLFIRINOX is associated with favorable outcomes for patients with resectable disease, BRPC, and LAPC.

In our study, patients who received long-duration neoadjuvant FOLFIRINOX and underwent surgery had a median OS of 41 months, which compares favorably with other reported results. In the SWOG1505 study, patients with resectable PDAC were randomized to six cycles of neoadjuvant FOLFIRINOX versus three cycles of neoadjuvant gemcitabine/nab-paclitaxel, a median OS of 23.2 months was reported for the perioperative FOLFIRINOX arm.^19^ In the Alliance A021501 trial, where patients received eight cycles of neoadjuvant FOLFIRINOX for BRPC, the median OS was 29.8 months.^10^ However, a higher median OS of 54.4 months was reported in the adjuvant FOLFIRINOX arm (12 cycles) of the PRODIGE-24 study; this arm comprised a highly select patient group (patients ≤79 years of age, a WHO performance score of 0 or 1, no significant cardiovascular disease, and a serum CA19-9 level of < 180 kU/L).^4^

Our institutional approach of using long-duration neoadjuvant chemotherapy is based on favorable early experience^9^ and the unclear added benefit of radiotherapy to chemotherapy for PDAC in the neoadjuvant and adjuvant setting. Results from several randomized trials suggest that radiotherapy provides no added benefit to chemotherapy in the neoadjuvant setting. In the Dutch randomized phase III PREOPANC trial, patients with resectable disease and BRPC were randomized to neoadjuvant chemoradiation versus upfront surgery, and there was no significant difference in OS.^7^ In the Alliance A021501 phase II trial, patients with BRPC were randomized to neoadjuvant FOLFIRINOX with or without radiotherapy followed by four cycles of postoperative FOLFOX6. The median OS was 29.8 months for the chemotherapy-only arm and 17.1 months for the chemotherapy plus preoperative radiotherapy arm.^10^ In the ESPAC5 phase II trial, patients with BRPC who received neoadjuvant chemotherapy (FOLFIRINOX or gemcitabine) had better 1-year OS than patients who had upfront surgery; however, those who received neoadjuvant chemotherapy with radiation did not.^11^ Finally, for patients with LAPC, the CONKO-007 phase III randomized trial examined the addition of radiotherapy to chemotherapy and failed to meet the primary endpoint of R0 resection rate, although the authors did report an improved resection rate without a concomitant improvement in OS.^12^

Although current guidelines recommend neoadjuvant therapy for patients with BRPC or LAPC, the role of neoadjuvant therapy for resectable disease is evolving. In the SWOG1505 phase II trial, there were no differences in 2-year OS (47% vs. 48%) or median OS (23.2 months vs. 23.6 months).^8^ In the SWOG1505 trial, 49% of patients completed all therapy, mainly due to the inability to receive adjuvant chemotherapy, and 88% of patients were able to complete neoadjuvant therapy.^8^ Based on these findings and the PRODIGE-24 study^4^ in which only two-thirds of patients completed adjuvant chemotherapy, the authors raised the question of whether total neoadjuvant therapy should be considered in future clinical trials.^8^ Our findings support this strategy; our patients completed a median of 10 cycles of FOLFIRINOX prior to surgery.

Recently, a large retrospective study using the National Cancer Database found that for patients with resectable PDAC, multiagent neoadjuvant chemotherapy followed by resection was associated with better survival than upfront surgery.^20^ A recent meta-analysis comparing upfront resection with neoadjuvant therapy in resectable pancreatic cancer did not find an improvement in DFS or OS.^21^ In the ongoing Alliance A021806 phase III trial, patients with resectable disease were randomized to eight cycles of neoadjuvant FOLFIRINOX followed by four cycles of adjuvant FOLFIRINOX versus 12 cycles of adjuvant FOLFIRINOX. The primary endpoint of this trial was two-year OS.^22^ While we await the results of this trial, the findings from our study provide insight into the favorable outcomes of long-duration neoadjuvant FOLFIRINOX for PDAC.

Our study has some limitations. First, because this was a retrospective analysis from a single-institution, our study is subject to referral and selection bias. All patients included in this study underwent surgery, so this is a highly selected group. Second, because the total number of patients who received long-duration neoadjuvant chemotherapy is not known, we could not calculate the resection rate. On the other hand, an important strength of our study was the ability to capture granular data regarding clinicopathological features that may not be available in large databases.

## Conclusion

Although neoadjuvant therapy is commonly used for PDAC, the routine use of long-duration therapy (median cycles: FOLFIRINOX = 10; gemcitabine-based = 7) is unique. Most (85%) patients received FOLFIRINOX; the R0 resection rate was 76%. Median OS was 41 months and did not differ significantly among patients with resectable disease, BRPC, or LAPC. This study shows that favorable surgical outcomes can be attained after long-duration neoadjuvant FOLFIRINOX therapy alone.
